# Ureteral Stenting after Uncomplicated Ureteroscopy for Distal Ureteral Stones: A Randomized, Controlled Trial

**DOI:** 10.1155/2014/892890

**Published:** 2014-11-09

**Authors:** Y. El Harrech, N. Abakka, J. El Anzaoui, O. Ghoundale, D. Touiti

**Affiliations:** Department of Urology, Hospital Avicenne, Marrakech 40000, Morocco

## Abstract

*Objectives*. We compared outcome and complications after uncomplicated ureteroscopic treatment of distal ureteral calculi with or without the use of ureteral stents. *Materials and Methods*. 117 patients, prospectively divided into three groups to receive a double j stent (group 1, 42 patients), ureteral stent (group 2, 37 patients), or no stent (group 3, 38 patients), underwent ureteroscopic treatment of distal ureteral calculi. Stone characteristics, operative time, postoperative pain, lower urinary tract symptoms (LUTS), analgesia need, rehospitalization, stone-free rate, and late postoperative complications were all studied. *Results*. There were no significant differences in preoperative data. There was no significant difference between the three groups regarding hematuria, fever, flank pain, urinary tract infection, and rehospitalisation. At 48 hours and 1 week, frequency/urgency and dysuria were significantly less in the nonstented group. When comparing group 1 and group 3, patients with double j stents had statistically significantly more bladder pain (*P* = 0.003), frequency/urgency (*P* = 0.002), dysuria (*P* = 0.001), and need of analgesics (*P* = 0.001). All patients who underwent imaging postoperatively were without evidence of obstruction or ureteral stricture. *Conclusions*. Uncomplicated ureteroscopy for distal ureteral calculi without intraoperative ureteral dilation can safely be performed without placement of a ureteral stent.

## 1. Introduction

Nowadays ureteroscopy has become the treatment of choice for managing ureteral stones, specially mid and distal ones [1].

There is no consensus on placing a ureteral catheter after uncomplicated ureteroscopy and it is still controversial. It is a routine justified by the belief that this practice decreases ureteral stricture formation, protects the kidney, and minimizes postoperative pain [2]. However the use of stents is accompanied by significant morbidity, including pain, infection, and irritative voiding symptoms [3, 4].

The aim of our study was to assess the need for routine ureteral stenting after uncomplicated ureteroscopic stone removal, evaluating the patient characteristics, stone features, complications, and treatment outcome among stented and unstented patients.

## 2. Materials and Methods

From January 2009 to January 2011, a total of 117 patients treated with successful ureteroscopy for distal ureteral stones were prospectively randomized to 3 groups. Group 1 included 42 patients in whom a double j stent was inserted, group 2 comprised 37 in whom a ureteral stent was inserted, and group 3 included 38 patients in whom no stent was inserted after stone removal.

Informed consent was obtained from all patients. Patients were excluded from the study when stone size was greater than 2 cm.

The mean age was, respectively, 44.1, 39.6, and 43.2 for group 1, group 2, and group 3.

Preoperative imaging consisted of KUB and renal ultrasonography with NCCT or IVP.

All procedures were performed under epidural anesthesia after discussion with patients.

Ureteroscopy was done with a 7.5 Fr semirigid ureteroscope. One 0.038-inch guide wire was inserted via cystoscopy under fluoroscopic guidance. The cystoscope was removed and a semirigid ureteroscope was passed into the ureter over the working guide wire with nonprior ureteral dilation. The pneumatic lithoclast (Swiss LithoClast) was used to fragment the offending calculus into pieces in all cases requiring lithotripsy.

The stents used in the study were 7 Fr in diameter.

In the patients of group 2, the ureteral stent was left for 24 hours. All patients in group 1 were rehospitalized after 3 weeks for double j stent removal.

All patients were closely followed up.

Procedures were considered a success if either a solitary calculus was removed in its entirety or all fragments were absent on radiographic followup. Radiographic followup typically consisted of a plain radiograph and renal ultrasound 1 month after the procedure.

Age, gender, stone characteristics, total operating time, the mean operative time, and mean hospital stay were all recorded.

For postoperative symptoms and the complications, a special questionnaire and a precise clinical exam were used, looking for flank pain, hematuria, dysuria, urgency, fever, and urinary tract infection. Postoperative pain and dysuria were measured using a 10 cm visual analog scale.

We also studied, the need of analgesics in postoperative time, the rate of rehospitalisation, and the appearence of an ureteral stricture.

Patients were followed up postoperatively with a minimum of 3 months (the mean follow-up was 12 months).

Differences in percentages (qualitative variables) were analyzed by the *χ* ^2^ test. Kruskal-Wallis and Mann-Whitney  *U* test were used for comparison of treatment groups. Statistical analyses were performed using the SPSS 15.0. The *P* value < 0.05 was considered statistically significant.

## 3. Results

The 3 study groups were comparable regarding patients and stone characteristics (Table 1). Overall mean stone size in the study was 8.7 ± 3.1 mm. Moreover, the ureteroscopy technique, type of intracorporeal lithotripsy, and method of stone retrieval were not significantly different between the treatment groups.

Ureteroscopy was successfully achieved in the three groups and the stone-free rate at 4 weeks was 100% in each group.

Mean operative time plus or minus standard deviation in group 1 was 36 ± 4 minutes, 34 ± 5 in group 2, and 31 ± 9 in group 3. Thus, operative time was not significantly longer when a ureteral stent or double j stent was placed.

Table 2 shows the mean visual analog pain scores at 48 hours and 1 week after ureteroscopy and other postoperative symptoms and complications in the three groups.

At 48 hours and 1 week, symptoms like frequency/urgency, dysuria were significantly less in the non stented group comparing to other groups.

When comparing group 1 and group 3, patients with double j stents had statistically significantly more bladder pain (*P* = 0.003), frequency/urgency (*P* = 0.002), dysuria (*P* = 0.001), and need of analgesics (*P* = 0.001) compared to those without stents.

There was no significant difference between the three groups regarding hematuria, fever, flank pain, urinary tract infection, and rehospitalisation.

When taking into consideration all LUTS and the need of analgesics, they were significantly more important in the first group using a double pigtail stent (group 1 versus group 2: *P* = 0.002; group 1 versus group 3: *P* = 0.001).

Readmission to the hospital for unremitting pain was necessary in 1 of 117 patients. No patients in the stented group required postoperative readmission to the hospital.

All complications were easily and successfully managed by conservative measures.

All patients (75%) who underwent imaging postoperatively were without evidence of obstruction or ureteral stricture on followup imaging.

## 4. Discussion

Ureteral stents are commonly placed after ureteroscopic stone extraction. The rationale for the routine use of ureteral stents after ureteroscopy originates from supposition, rather than from evidence-based medicine. However, the placement of a ureteral stent may be associated with significant morbidity.

Joshi et al. identified patient morbidity associated with ureteral stents as a significant health problem and investigated it in detail [5]. They found that ureteral stents are associated with significant symptoms, such as pain affecting daily activities (80%), urinary symptoms (73%), and reduced work capacity (58%), which reduce quality of life. During the research, it became clear that stents profoundly affect physical and psychosocial health and have a negative impact on functional capacity and work performance [3, 5].

In our present study involving 117 patients, we did not find significant difference between the three groups concerning flank pain, hematuria, fever, and urinary tract infection.

Several trials have demonstrated similar postoperative complication rates among stented and unstented patient populations. These trials have several similarities with our study including a preponderance of patients with distal ureteral calculi, uncomplicated procedure, and absence of ureteral dilation.

In the study of Ibrahim et al. [6], early postoperative complications, including low grade fever, hematuria, and urinary tract infection, were observed in 22 patients (20%) in nonstented patients and 19 (19%) in stented ones, a difference of no significant value. Mean initial hospitalization and time to return to normal physical activity were not different between the 2 groups. At 48 hours and 1 week there was no significant difference in flank pain between the 2 groups.

Borboroglu et al. [7] in a multi-institutional prospective randomized controlled study were surprised to find a statistically significant lower incidence in flank pain in their patients without stents and explained it by the fact that perhaps the patients with stents had so much overall pain that they reflected this in response to the flank pain question.

The main significant difference that was found in our study concerned dysuria, bladder pain, frequency, and the need of analgesics after the ureteroscopy.

Nabi et al. [8] reported the results of a meta-analysis of 9 randomized, controlled trials of stenting following uncomplicated ureteroscopy. The incidence of lower urinary tract symptoms was significantly higher in participants who had a stent inserted after ureteroscopy.

The same results were confirmed by Ibrahim et al. [6] and Borboroglu et al. [7] who found that patients without stents had significantly less bladder pain, urinary symptoms, and narcotic use postoperatively.

In a recent meta-analysis [9] studying the effect of ureteral stent placement on postureteroscopy complications, the authors concluded that the published evidence supports the practice of omitting a ureteral stent after an uncomplicated ureteroscopic procedure. This meta-analysis attests that there is a slightly lower absolute risk of complication associated with the placement of a ureteral stent after ureteroscopy but does not detect a significant difference in outcome between patients who undergo ureteral stent placement after ureteroscopy and those who do not.

In the stented group, a cystoscopy is needed to remove the double pig tail stent. Richter et al. [10] claimed that placement of a ureteral stent is “a friendly procedure with unfriendly high morbidity.” The additional cystoscopy for stent removal is a cause of discomfort and overall cost.

Netto Jr. et al. [11] assessed the cost-effectiveness of routine ureteral stenting after ureteroscopic stone removal. The cost per patient in patients with a stent was $9,900.95 versus $3,661.78 for patients without, about 30% the cost of using a stent. They concluded that routine catheter placement after ureteroscopic stone removal increased operative time and the cost of procedure and did not seem to improve patient outcome.

Some authors [12] sought to identify significant clinical characteristics affecting postoperative morbidity in unstented patients. They found that patients undergoing bilateral stentless ureteroscopy, those with recent or recurrent urinary tract infections, and those with a history of urolithiasis are at greater risk for a postoperative complication. Other factors such as operative time and renal stone location may have a role as well. This will help to select patients suitable for stentless procedure.

An advantage of the current series is the prospective and randomized comparison of three arms. To our knowledge, there have been no published studies of outcomes and results among three different groups including patients with double j stent, ureteral stent, and no stent.

The current study has some limitations, including the absence of a validated symptom score for proper assessment of pain and lower urinary tract symptoms. Another limitation is the absence of a precise assessment of the amount of analgesia used and the absence of assessment of cost.

## 5. Conclusion 

The ureteral stent has become an integral part of the urological armamentarium. However, stent related morbidity is a reality in the majority of patients.

Uncomplicated ureteroscopy for distal ureteral calculi without intraoperative ureteral dilation can safely be performed without placement of a ureteral stent.

Patients without stents had significantly less pain, fewer urinary symptoms, and decreased analgesic use postoperatively and are not at risk of increased late complications.

The other potential benefits to leaving patients without stents after ureteroscopy are cost savings and the avoidance of followup cystoscopy for stent removal.

## Figures and Tables

**Table 1:** Patient's characteristics and results.

	Double j stent (group 1, 42 patients)	Ureteral stent (group 2, 37 patients)	No stent (group 3, 38 patients) stent	*P*
Mean patient age (y, range)	44.1 (22–72)	39.6 (27–72)	43.2 (20–76)	0.24
Side of stone: R/L	16/26	17/20	20/18	0.12
Mean stone size (mm)	8.6 ± 3.4	10.1 ± 2.7	9.6 ± 3.6	0.18
Mean operative time (min)	36 ± 4	34 ± 5	31 ± 9	0.17
Success rate (%)	100	100	100	0.69

**Table 2:** Postoperative symptoms and complications.

	Double j stent (group 1; 42 patients)	Ureteral stent (group 2; 37 patients)	No stent (group 3; 38 patients) stent	*P*
Mean flank pain score at 48 hours (0–10) ± S.D.	4.3 ± 2.1	4.3 ± 2.3	4.7 ± 1.9	0.14
Mean flank pain score on day 7 (0–10) ± S.D.	2.6 ± 1.4	2.1 ± 1.2	2.1 ± 1.4	0.09
Mean bladder pain score at 48 hours (0–10) ± S.D.	5.3 ± 2.6	4.9 ± 1.9	2.2 ± 1.4	0.004
Mean bladder pain score on day 7 (0–10) ± S.D.	4.8 ± 2.5	2.5 ± 1.7	1.9 ± 1.1	0.003
Dysuria number (%)	11 (26.1)	8 (21.6)	5 (13.1)	0.002
Hematuria number (%)	3 (7.1)	3 (8.1)	2 (5.2)	0.67
Frequency/urgency number (%)	17 (40.4)	10 (27.0)	7 (18.4)	<0.001
Need of analgesics in follow-up	11 (26.1)	4 (10.8)	3 (7.8)	<0.001
Fever number (%)	3 (7.1)	2 (5.4)	3 (7.8)	0.50
Urinary tract infection number (%)	3 (7.1)	2 (5.4)	3 (7.8)	0.39
Rehospitalization number (%)	0	0	1	0.34
Ureteral stricture number (%)	0	0	0	0.70
Mean hospital stay (hours)	26	26	25	0.48
